# First molecular detection of *Theileria haneyi* infection in horses in Southern Spain

**DOI:** 10.3389/fvets.2026.1841333

**Published:** 2026-06-04

**Authors:** Francisco J. Mendoza, Alejandro Pérez-Écija, Carlos E. Suarez, Esther Martínez, Ana Navarro, Lowell S. Kappmeyer, Reginaldo G. Bastos

**Affiliations:** 1 Department of Animal Medicine and Surgery, College of Veterinary Medicine, University of Cordoba, Cordoba, Spain; 2 Department of Veterinary Microbiology and Pathology, College of Veterinary Medicine, Washington State University, Pullman, WA, United States; 3 Gasset Laboratory, DAV Salud Group SL, Granada, Spain; 4 Animal Disease Research Unit, Agricultural Research Service, United States Department of Agriculture (USDA), Pullman, WA, United States

**Keywords:** equid, parasitology, piroplasmosis, *Theileria equi*, *Theileria haneyi*

## Abstract

**Background:**

Equine piroplasmosis (EP) is a tick-borne disease of equids caused by the hemoparasites *Theileria equi, Babesia caballi*, and *Theileria haneyi*. *T. haneyi*, a close relative of *T. equi* that causes mild clinical signs under experimental conditions, has been reported in several countries, adding complexity to EP ecology, diagnosis, and treatment. However, its presence in Spain remains unknown, which is concerning given the country's importance in equine genetics and international trade. Hereby, we investigated the presence of *T. haneyi* in blood samples from equids in Spain.

**Methods:**

Blood samples from 222 equids (218 horses, two donkeys, two mules) suspected of EP across nineteen Spanish provinces were analyzed. DNA was extracted using standard protocols. *T. haneyi* was detected by nested PCR targeting a hypothetical protein gene (GenBank MT896770.1), and *T. equi* by nested PCR targeting the EMA1 gene. For *T. haneyi*-positive samples, the EMA11 gene was amplified and sequenced, and nucleotide alignments were generated using ClustalW.

**Results:**

Three horses out of 222 (3/222; 1.35%) were positive for *T. haneyi*. These three positive horses were located in Southern Spain in the provinces of Huelva, Cadiz, and Malaga. Additionally, 76 (76/222; 34.2%) were positive for *T. equi*. All three *T. haneyi*-positive horses were also found PCR-positive for *T. equi*. Alignment analysis of a 209-nucleotide sequence from the EMA11 gene revealed a high level of identity between the Spanish and the *T. haneyi* reference isolate U.S. Eagle Pass sequences. One nucleotide change, representing a silent mutation, was detected in one of the *T. haneyi*-positive samples, while the other two showed 100% nucleotide identity.

**Conclusion:**

The results indicate the first detection of *T. haneyi* in horses in Spain. These findings have important implications for equine clinicians, regulatory agencies, and animal health surveillance programs not only in Spain but also in other countries with significant equine industries, particularly in those European countries within the Schengen area. A systematic epidemiological study should be now undertaken to determine the prevalence and geographic distribution of *T. haneyi* infections in Spain.

## 1 Introduction

Equine piroplasmosis (EP) is an economically significant disease of equids caused by the apicomplexan hemoprotozoans *Babesia caballi, Theileria equi* and the recently discovered *Theileria haneyi* affecting horses, donkeys, and mules (1). These parasites are transmitted by several genus of the *Ixodidae* tick family or through iatrogenic means (1). This parasitic disease is globally distributed and causes significant economic losses, both directly, through clinical signs such as poor performance, abortions, and acute hemolysis, and indirectly, through international movement restrictions on infected animals that limit their purchase or participation in events such as sport competitions, shows, and auctions (2).

Although different clinical presentations can be observed (3, 4), chronic infection, particularly by *T. equi* or *T. haneyi*, in asymptomatic carriers is the most common clinical form in endemic regions due to still unknown immune-evasion mechanisms that prevent the host from clearing the parasites, combined with the low efficacy of currently available treatments (5–8). In addition, a previous study showed that co-infection with *T. haneyi* appears to reduce the imidocarb dipropionate efficacy against *T. equi* (6), which is of concern considering that co-infection between *T. haneyi* and *T. equi* is common in endemic areas (9).

Equine piroplasmosis is endemic in many European countries, such as Spain, France, Italy, and Portugal (10). Because of the high demand for Spanish Purebred horses, Spain is a major international exporter, primarily to the USA, Mexico, and South American countries, as well as to other parts of Europe or China (11). Spanish Purebred horses can be transported freely within the Schengen area, which may allow the movement of EP-infected horses without any restrictions throughout Europe. Although *T. haneyi* has been previously reported in several countries (12–17), no data is currently available regarding the presence of *T. haneyi* in Spain. Since *T. haneyi* infection is primarily asymptomatic, and given Spain's crucial role in the equine industry, investigating the presence of this parasite in the country is important. Therefore, the aims of this study were to evaluate the presence of *T. haneyi* in equids with clinical suspicion of piroplasmosis in Spain and to check for the possibility of co-infections with both *T. haneyi* and *T. equi*.

## 2 Materials and methods

### 2.1 Animal sampling

Blood samples submitted for PCR analysis to a national private veterinary reference laboratory (Gasset Laboratory, DAV Salud Group SL, Granada, Spain) from equids with suspicion of EP based on clinical signs (anorexia, poor performance, jaundice, weight loss, fever, etc.) during 2024 and 2025 were included in this study. Blood samples were shipped overnight and used for DNA extraction.

### 2.2 DNA extraction

DNA was extracted from 200 μL EDTA blood sample using an automatized nucleic acid extraction system (MagNA Pure 24 Instruments, Roche Diagnostics, Barcelona, Spain) following the manufacturer's instructions. The extracted DNA was stored at −20°C until used for subsequent analysis.

### 2.3 Nested PCR for *T. haneyi*

The presence of *T. haneyi* DNA in the equid blood samples was examined by nested PCR (nPCR) targeting a hypothetical parasite protein gene (GenBank accession number: MT896770.1) which is absent in the *T. equi* genome (12). As previously reported (6–8, 12, 13, 18), this technique and protocol have been able to efficiently detect *T. haneyi* in acutely and chronically infected horses. Briefly, reactions were performed in 20-μL total volume using the DreamTaq™ Hot Start Green PCR Master Mix (Thermo Fisher Scientific, Waltham, MA, USA). For the first round of amplification, 5 μL of template gDNA and 10 μM of the following external primers were used: 5′-CCATACAACCCACTAGAG-3′ and 5′-CTGTCATTTGGGTTTGATAG-3′ (amplicon size: 382 base pairs; Supplementary Table 1). Cycling conditions for the reaction consisted of a denaturation step of 95 °C for 4 min, followed by 35 cycles of denaturation for 20 s at 95 °C, annealing for 30 s at 63.5 °C, and extension for 20 s at 72 °C. These steps were followed by a final extension for 10 min at 72 °C. Second round of amplification was performed using 1 μL of the first reaction and 10 μM of the following internal primers: 5′-GACAACAGAGAGGTGATT-3′ and 5′-CGTTGAATGTAATGGGAAC-3′ (amplicon size: 238 base pairs; Supplementary Table 1). Cycling conditions for this second reaction consisted of a denaturation step of 95 °C for 4 min, followed by 35 cycles of denaturation for 20 s at 95 °C, annealing for 30 s at 58.1 °C, and extension for 20 s at 72 °C. These steps were followed by a final extension for 10 min at 72 °C. PCR product was analyzed by agarose gel using standard protocols. A positive control, consisting of DNA extracted from an experimentally *T. haneyi*-infected horse, and a non-template negative control were used for the nPCR, as previously described (8, 12, 13, 18). Positive samples were run by replicates from a new blood sample collected days later.

### 2.4 PCR amplification of EMA11 *T. haneyi* gene and sequence alignment

Amplification of the full-length EMA11 *T. haneyi* gene was conducted via PCR as previously described (12) from *T. haneyi*–positive samples using the following primers: 5′-ATGTTGGCTAGGTCTTTTGT-3′ and 5′-GTAAAAGAGAGTAGAGAAAGCAA-3′ (amplicon size: 825 base pairs; Supplementary Table 1). Cycling conditions for the reaction consisted of a denaturation step of 95 °C for 4 min, followed by 40 cycles of denaturation for 20 s at 95 °C, annealing for 30 s at 55.0 °C, and extension for 20 s at 72 °C. These steps were followed by a final extension for 10 min at 72 °C. PCR product was analyzed by agarose gel using standard protocols. A positive control, consisting of DNA extracted from an experimentally *T. haneyi*-infected horse, and a non-template negative control were used for the EMA11 PCR. Briefly, EMA 11 amplicons sequences were analyzed and trimmed to remove low-quality bases from the 5′ and 3′ ends. The resulting high-quality 210-bp segment of each positive sample was used for nucleotide alignment analyses in comparison with the reference isolate U.S. Eagle Pass of *T. haneyi* using ClustalW software (19, 20). An additional alignment using the EMA11 segment of the Spanish *T. haneyi* sequences, the Eagle Pass *T. haneyi* reference isolate, and the *T. equi* EMA1, the prototype representative of the EMA gene family (21), was also performed as described above.

### 2.5 Nested PCR for *T. equi*

Detection of *T. equi* DNA in the equid blood samples was performed by nPCR targeting EMA1, as previously described (21). Reactions were carried out in 20-μL total volume using the DreamTaq™ Hot Start Green PCR Master Mix (Thermo Fisher Scientific, Waltham, MA, USA). The first round of amplification was performed using 2 μL of gDNA as template and 10 μM of the following external primers: 5′-GAGGAGGAGAAACCCAAG-3′ and 5′-GCCATCGCCCTTGTAGAG-3′ (amplicon size: 567 base pairs; Supplementary Table 1). Cycling conditions for the reaction consisted of a denaturation step of 95 °C for 5 min, followed by 35 cycles of denaturation for 20 s at 95 °C, annealing for 20 s at 60 °C, and extension for 20 s at 72 °C. These steps were followed by a final extension for 10 min at 72 °C. Second round of amplification was performed using 1 μL of the first reaction as template and 10 μM of the following internal primers: 5′-TCAAGGACAACAAGCCATAC-3′ and 5′-TTGCCTGGAGCCTTGAAG-3′ (amplicon size: 229 base pairs; Supplementary Table 1). Cycling conditions for this second reaction consisted of a denaturation step of 95 °C for 5 min, followed by 35 cycles of denaturation for 5 s at 95 °C, annealing for 5 s at 60 °C, and extension for 5 s at 72 °C. These steps were followed by a final extension for 10 min at 72 °C. PCR product was analyzed by agarose gel using standard protocols. DNA from experimentally *T. equi*-infected horses and non-template reaction were used as positive and negative controls, respectively.

### 2.6 PCR amplification of the *Theileria spp*. full length 18S gene and sequence alignment

PCR amplification of the *Theileria spp*. full length 18S gene was performed using the following primers (Supplementary Table 1): 5′-AAGCCATGCATGTCTAAGTATAAGCTTT-3′ and 5′-GAATAATTCACCGGATCACTCG-3′ (12). Cycling conditions consisted of a denaturation step of 95 °C for 5 min, followed by 40 cycles of denaturation for 5 s at 95 °C, annealing for 1 min at 60 °C, and extension for 1 min at 72 °C. These steps were followed by a final extension for 10 min at 72 °C. PCR product was analyzed by agarose gel using standard protocols. The 18S amplicon was sequence by Eurofins Genomics (Louisville, KY, USA).

## 3 Results

### 3.1 Study population

A total of 222 blood samples of equids (218/222 horses, 2/222 donkeys, and 2/222 mules) were submitted to our reference laboratory for EP PCR analysis during the study period. Samples in this study were collected from 19 provinces of Spain: Alicante, Almeria, Avila, Badajoz, Barcelona, Cadiz, Castellon, Cordoba, Granada, Huelva, Jaen, Madrid, Malaga, Murcia, Navarra, Sevilla, Teruel, Valencia, and Zamora.

### 3.2 Molecular Detection of *T. haneyi* and *T. equi*

**Figure 1:**
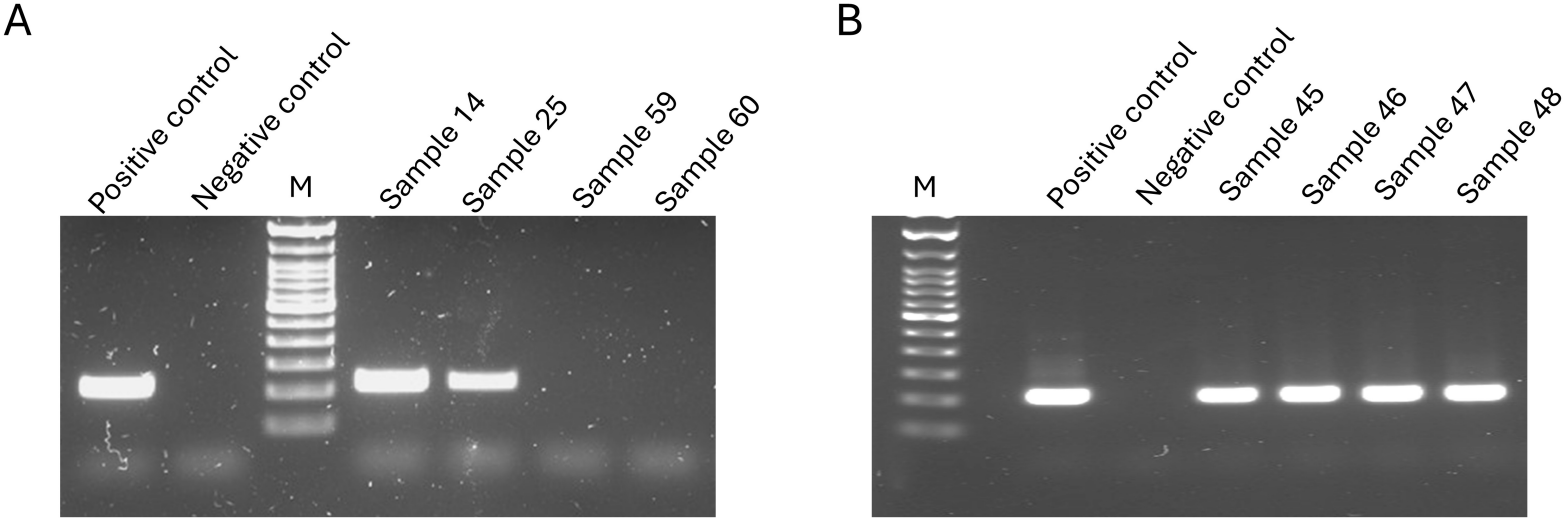
Representative agarose gel showing two *T. haneyi* positive samples (samples 14 and 25) and two negative (samples 59 and 60) horses by nested PCR **(A)**. Representative agarose gel showing four *T. equi* positive samples (samples 45–48) **(B)**.

**Figure 2:**
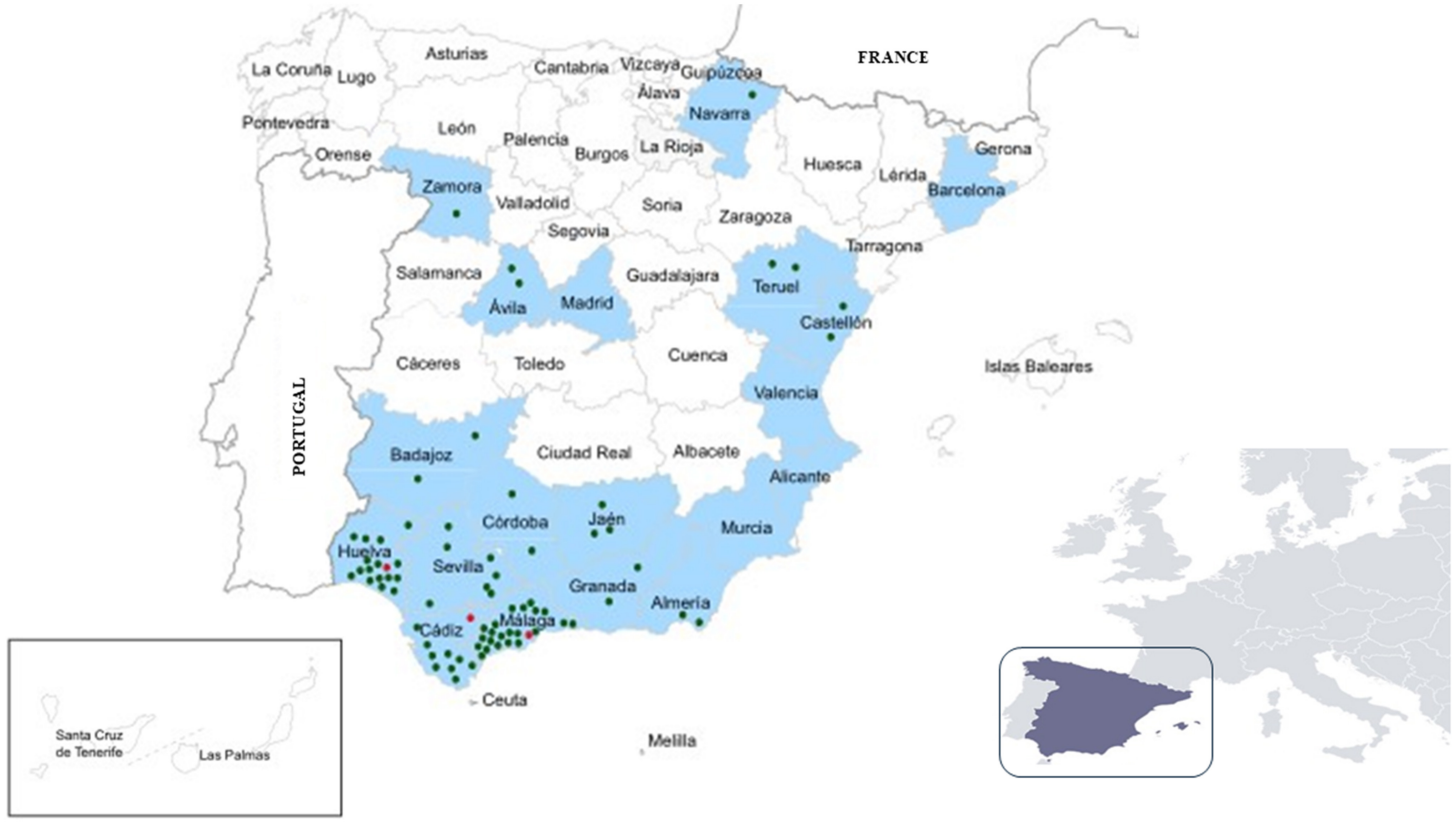
Map of Spain showing the provinces with nested PCR positive horse samples for *T. haneyi* (*n* = 3, red dots) and *T. equi* (*n* = 76, green dots). Blue areas represent those districts from where blood samples were submitted.

Three horses out of 222 samples (3/222; 1.35%) analyzed were positive for the *T. haneyi*-specific nPCR (Figure 1A). All three horses positive for *T. haneyi* were also co-infected with *T. equi* (Table 1). These animals were in three different provinces located in Southern Spain: Huelva (*n* = 1), Cadiz (*n* = 1), and Malaga (*n* = 1) (Figure 2). Seventy-six out of 222 samples (76/222, 34.2%; 75/76 horses and 1/76 donkey) were positive for the *T. equi* nPCR (Figure 1B).

**Table 1:** Signalment of the *T. haneyi* positive equids.

Sample ID	Species	Gender	Age (years old)	Breed	District (Province)
14	Horse	Stallion	4	Marismeña	Huelva
25	Horse	Gelding	8	Anglo-Arabian	Cadiz
135	Horse	Gelding	14	Marismeña	Malaga

### 3.3 Alignments analysis of *T. haneyi* EMA11, *T. equi* EMA1, and *Theileria spp*. full length 18S

Results demonstrated a high level of nucleotide identity between the *T. haneyi* EMA11 amplicons from the Spanish samples and the reference U.S. Eagle Pass isolate (Table 2). In fact, two of the Spanish amplicons (samples #25 and #135) were identical to the Eagle Pass reference isolate. The Spanish sample #14 amplicon showed a single nucleotide alteration (guanine by adenine) at the position 204 in comparison to the Eagle Pass *T. haneyi* isolate (Supplementary Figure 1). This nucleotide change was a silent mutation resulting in no alteration of the coded amino acid.

**Table 2:** Percentage nucleotide identity among a segment of EMA11 (210 bp) from the Eagle Pass strain of the *T. haneyi* (reference isolate), EMA11 from the Spanish strain of the Spanish *T. haneyi* samples, and from the Florida strain of the *T. equi* EMA1 (prototype of the EMA gene family).

Target gene	*T. haneyi* Eagle Pass EMA11	*T. haneyi* EMA11 sample #14	*T. haneyi* EMA11 sample #25	*T. haneyi* EMA11 sample #135
*T. haneyi* Eagle Pass EMA11	100%	99.87%	100%	100%
*T. equi* Florida EMA1	63.25%	63.12%	63.25%	63.25%

Regarding alignment between the *T. haneyi* EMA11 from Spanish samples and EMA1 *T. equi* Florida strain (reference isolate), the Spanish samples #25 and #135 had a 63.25% of identity, which was similar to the nucleotide identity observed for the Eagle Pass *T. haneyi* strain (Table 2), while the sample #14 had 63.12% of identity (Table 2).

*Theileria spp*. full length 18S gene sequencing from the three horses infected with *T. haneyi* showed a pattern consistent with mixed infection, as expected given the co-infections with *T. equi*. Consequently, no further analysis of the *Theileria spp*. full length 18S sequences was performed.

## 4 Discussion

*Theileria haneyi* has been reported in horses in several countries worldwide, including equids in the Americas, Africa and Asia (12, 14, 17, 22–27). Recently, the parasite has been also detected in Europe, specifically in Italy and Portugal (15, 28). To date, no data on the presence of *T. haneyi* in Spain are available, despite of the prominent role the country plays in the global equine industry (11). In this study, we performed molecular analyses to investigate the presence of *T. haneyi* in blood samples from equids displaying clinical signs compatible with EP. Herein we report for the first time the presence of horses infected with *T. haneyi* in three provinces located in Southern Spain.

Scarce information is currently available on the global prevalence of *T. haneyi*. A recent study demonstrated a parasite prevalence of 11.76% in equids (horses and donkeys) randomly collected in various provinces of China (16). Researchers in South Africa detected *T. haneyi* DNA in 67.6% of a total of 170 horses tested (23). Another recent study demonstrated a prevalence of 9.5% in horses in Northern Italy (15). However, other studies performed in Nigeria and Brazil have reported lower true prevalences (17, 27). A limitation of our study was the low sample size taken into consideration the overall equid population in Spain. In addition, the study was designed using samples derived from equids with suspected EP in order to maximize the chances of detecting *T. haneyi* infection; thus, sampling was not representative of the equid population in Spain and did not cover the entire national territory. Consequently, an epidemiological analysis could not be performed, and further studies are warranted in order to assess the true prevalence and geographic distribution of *T. haneyi* in Spain. Moreover, experimental studies have demonstrated that *T. haneyi* infection causes minor clinical signs during the acute phase of the infection, evolving to chronicity (6, 12, 13, 18). Therefore, due to the absence of clinical manifestations, these horses were not tested in our study.

*T. haneyi*–infected animals are primarily asymptomatic carriers due to low parasite‘s pathogenicity (12, 13), serving as silent reservoirs that enable continuous reinfection of ticks. Scarce information is currently available regarding tick species capable of transmitting *T. haneyi*. Recently, it was reported that *Haemaphysalis longicornis* was unable to transstadially transmit *T. haneyi* to horses (29). Whether the distribution and/or presence of specific tick species in different countries is a factor influencing the presence and distribution of *T. haneyi* is currently unknown. In this regard, previous reports have demonstrated that differences in climate and soil characteristics, which support suitable ecosystems, partly explain the variations in *T. equi* and *B. caballi* prevalence among countries (30, 31).

The three *T. haneyi-*positive horses in our study were housed in Andalusia, the southernmost region in Spain and Europe. Andalusia is the area with more registered horses (approximately 32%) and farms (approximately 39%) in Spain (32). This region plays a major role in the maintenance of the EP endemic status in Spain (11). These findings highlight the potential impact that horses residing in this area may have on international horse trading, either for commercial purposes or for international movement. Another limitation of this study was that most blood samples were submitted from Andalusia. Therefore, additional studies incorporating a larger number of samples from other Spanish regions, including those provinces not tested (19 out of 50), would improve the likelihood of detecting *T. haneyi*-positive horses in other provinces of the country.

Another limitation of our study was the absence of phylogenetic tree analysis to further compare with those previously described *T. haneyi* strain worldwide. Although the full length 18S was amplified and sequenced in the three *T. haneyi* positive Spanish horses, the amplicons had a poor quality for performing phylogenetic analysis, since the three horses were also co-infected with *T. equi*, which also generated amplicons due to primer cross-reactivity in the PCR reaction. However, despite full genome sequence has been recently reported for Eagle Pass *T. haneyi* strain (19), given the novelty of this species, other loci sequences remain poorly defined. In addition, although other EMA genes (e.g., EMA10, EMA12, EMA13) could be unique for *T. haneyi* (12), it might not be conservative among some *T. haneyi* strains (e.g., U.S. Eagle Pass, Spain, China, and other countries). Thus, additional studies are necessary to evaluate whether *T. haneyi* strains expressing different EMA genes are circulating in equids in Spain, and whether the *T. haneyi* strain detected in Spain could be allocated within clade C (*T. haneyi-*like group) (33, 34). Although a previous phylogenetical analysis in Spain identified only clades A, D, and E (35).

Although only a 210 bp fragment of the *T. haneyi* EMA11 gene was evaluated in this study, the aligned region revealed high nucleotide identity with the reference isolate. This finding is particularly important because the EMA11 protein is unique to *T. haneyi* and therefore absent in *T. equi*, and it has been previously tested and used in serological assays (12). The nucleotide sequence similarity and functional conservation of the EMA gene family within *Theileria* species are remarkable despite their speciation (36). Notably, the adenine nucleotide at position 204 is conserved in EMA11 of the reference *T. haneyi* Eagle Pass isolate, and also in Spanish samples #25 and #135, and in EMA1 of *T. equi* (Florida strain). In contrast, this adenine was substituted by a guanine in the Spanish sample #14. Despite the substitution, the mutation appears to be silent and does not alter the encoded amino acid.

In conclusion, these results provide the first molecular evidence of *Theileria haneyi* infection in horses in Spain, demonstrating that actively infected animals are circulating in the country. The data reinforce that a serological surveillance should be performed in Spain to establish an updated map of seropositive equids for *T. haneyi*, along whole national territory, to establish adequate transboundary measures and control programs.

## Data Availability

The original contributions presented in the study are included in the article/Supplementary material, further inquiries can be directed to the corresponding author/s.
