# Supplementary material for: First molecular detection of Theileria haneyi infection in horses in Southern Spain
Source: Front Vet Sci. 2026 Jun 4;13:1841333. doi: 10.3389/fvets.2026.1841333 (PMC13275341; doi:10.3389/fvets.2026.1841333)
Supplement: Supplementary file 1 [file Table_1.docx]

**Supplementary Table 1. Primer sequences used in this manuscript for molecular detection of *Theileria equi and Theileria haneyi***

| ***Species*** | ***Primer*** | ***Target***  ***Gene*** | ***Amplicon size***  ***(bp)*** | ***Sequence 5'-3'*** | ***Reference*** |
| --- | --- | --- | --- | --- | --- |
| ***T. equi*** | nPCR ExtFor | EMA1 | 567 | GAGGAGGAGAAACCCAAG | 21 |
|  | nPCR ExtRev |  |  | GCCATCGCCCTTGTAGAG |  |
|  | nPCR IntFor |  | 229 | TCAAGGACAACAAGCCATAC |  |
|  | nPCR IntRev |  |  | TTGCCTGGAGCCTTGAAG |  |
| ***T. haneyi*** | nPCR ExtFor | EMA11 | 382 | CCATACAACCCACTAGAG | 12 |
|  | nPCR ExtRev |  |  | CTGTCATTTGGGTTTGATAG |  |
|  | nPCR IntFor |  | 238 | GACAACAGAGAGGTGATT |  |
|  | nPCR IntRev |  |  | CGTTGAATGTAATGGGAAC |  |
| ***T. haneyi*** | EMA11F | Full length EMA11 | 825 | ATGTTGGCTAGGTCTTTTGT | 12 |
|  | EMA11R |  |  | GTAAAAGAGAGTAGAGAAAGCAA |  |
| **Theileria spp.** | 18SFor-T | Full length 18S | 1600 | AAGCCATGCATGTCTAAGTATAAGCTTT | 12 |
|  | 18SRev-T |  |  | GAATAATTCACCGGATCACTCG |  |
