# Supplementary material for: First molecular detection of Theileria haneyi infection in horses in Southern Spain
Source: Front Vet Sci. 2026 Jun 4;13:1841333. doi: 10.3389/fvets.2026.1841333 (PMC13275341; doi:10.3389/fvets.2026.1841333)
Supplement: Supplementary file 2 [file Image_1.pdf]

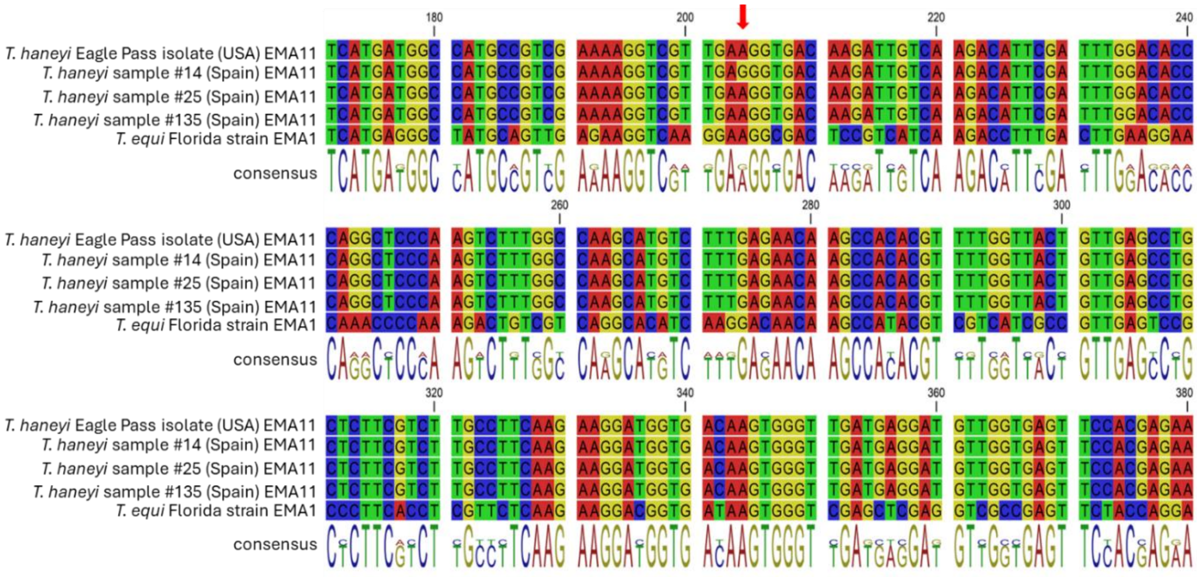

Supplementary Figure 1. Alignment of a 210-base pair segment of the *T. haneyi* merozoite antigen 11 (EMA11) from the Spanish *T. haneyi* isolates, EMA11 from the U.S. Eagle Pass reference isolate of *T. haneyi*, and EMA1 of *T. equi* Florida strain as the prototype for the EMA gene family. Red arrow at position 204 indicates the nucleotide alteration in the *T. haneyi* Spanish sample #14 compared to the Eagle Pass *T. haneyi* and the Spanish samples #25 and #135.
